# *Antrodia camphorata* Potentiates Neuroprotection against Cerebral Ischemia in Rats via Downregulation of iNOS/HO-1/Bax and Activated Caspase-3 and Inhibition of Hydroxyl Radical Formation

**DOI:** 10.1155/2015/232789

**Published:** 2015-08-25

**Authors:** Po-Sheng Yang, Po-Yen Lin, Chao-Chien Chang, Meng-Che Yu, Ting-Lin Yen, Chang-Chou Lan, Thanasekaran Jayakumar, Chih-Hao Yang

**Affiliations:** ^1^Department of Surgery, Mackay Memorial Hospital and Mackay Medical College, Taipei, Taiwan; ^2^Department of Pharmacology, School of Medicine, Taipei Medical University, Taipei, Taiwan; ^3^Cardiovascular Division, Department of Surgery, Yuan's General Hospital, Kaohsiung, Taiwan; ^4^Department of Cardiology, Cathay General Hospital, Taipei, Taiwan; ^5^Graduate Institute of Medical Sciences, College of Medicine, Taipei Medical University, Taipei, Taiwan; ^6^Sheen Chain Biotechnology, Co., Ltd., Taipei, Taiwan

## Abstract

*Antrodia camphorata* (*A. camphorata*) is a fungus generally used in Chinese folk medicine for treatment of viral hepatitis and cancer. Our previous study found *A. camphorata* has neuroprotective properties and could reduce stroke injury in cerebral ischemia animal models. In this study, we sought to investigate the molecular mechanisms of neuroprotective effects of *A. camphorata* in middle cerebral artery occlusion (MCAO) rats. A selective occlusion of the middle cerebral artery (MCA) with whole blood clots was used to induce ischemic stroke in rats and they were orally treated with *A. camphorata* (0.25 and 0.75 g/kg/day) alone or combined with aspirin (5 mg/kg/day). To provide insight into the functions of *A. camphorata* mediated neuroprotection, the expression of Bax, inducible nitric oxide synthase (iNOS), haem oxygenase-1 (HO-1), and activated caspase-3 was determined by Western blot assay. Treatment of aspirin alone significantly reduced the expressions of HO-1 (*P* < 0.001), iNOS (*P* < 0.001), and Bax (*P* < 0.01) in ischemic regions. The reduction of these expressions was more potentiated when rats treated by aspirin combined with *A. camphorata* (0.75 g/kg/day). Combination treatment also reduced apoptosis as measured by a significant reduction in active caspase-3 expression in the ischemic brain compared to MCAO group (*P* < 0.01). Moreover, treatment of *A. camphorata* significantly (*P* < 0.05) reduced fenton reaction-induced hydroxyl radical (OH^•^) formation at a dose of 40 mg/mL. Taken together, *A. camphorata* has shown neuroprotective effects in embolic rats, and the molecular mechanisms may correlate with the downregulation of Bax, iNOS, HO-1, and activated caspase-3 and the inhibition of OH^•^ signals.

## 1. Introduction

Stroke denotes to a rapid worldwide neurological impairment that victims may grieve paralysis and speech disorder, as well as loss of cognizance due to either ischemia or hemorrhage. It is considered as one of the leading causes of death and disability worldwide [1]. Currently, intravascular techniques and thrombolytic agents have remarkably decreased functional deficits. Although there are good improvements established in treatment, there is still little that can be done to prevent stroke-related brain damage. Therefore, active prevention and control of stroke are of great clinical value. Aspirin is the most widely used drug for the prevention of secondary stroke. However, the incidence of cerebral haemorrhage and other bleeding events are major issues, while recurrent stroke is controlled by this treatment [2]. Thus, research has been focused on finding alternative drugs that may act on different pathways that have been used to recover them from the group of inflammation, necrosis, and apoptosis, all of which are associated in ischemic stroke [3]. Natural products are a prolific source of bioactive agents of different structure and varying biological activities. In the search for neuroprotective agents from natural sources, a number of plant extracts and several natural products isolated from them have been reported to provide neuroprotection against ischemic stroke [4].

*Antrodia camphorata* is being used as the complementary and alternative medicines, and it grows only on the inner heartwood wall of the endangered species* Cinnamomum kanehirai* Hay (Lauraceae) [5–7].* A. camphorata* has long been used in Taiwanese folk medicine for abdominal pain, chemical intoxication, diarrhea, hypertension, itchy skin, and hepatoma [8]. Studies have demonstrated that* A. camphorata* induces significant apoptosis of human promyelocytic leukemia (HL-60) cells [9] and its extracts may be used as an adjuvant antitumor agent for human hepatoma cells, which are resistant to most other antitumor agents. Our previous study had shown that* A. camphorata* possesses antioxidant effects against carbon tetrachloride- (CCl_4_-) induced hepatic injury* in vivo*, via mediating free radical scavenging activities [10].* A. camphorata* also has shown to reduce H_2_O_2_-induced lipid peroxidation and enhance hepatic glutathione-dependent enzymes upon protecting CCl_4_-induced damage on rat liver [11]. Despite the fact that our very recent study has demonstrated that* A. camphorata* has neuroprotective effect against ischemic stroke in rats through reducing infarct volume and improves neurobehavioral scores and regulating blood perfusion without increasing hemorrhagic transformation [12], the molecular mechanism of action of* A. camphorata* in this effect is remained obscured. Thus, in this study, we investigated the effects and possible mechanisms of action of* A. camphorata* on ischemic stroke in rats.

## 2. Materials and Methods

### 2.1. Plant Material

Well Shine Biotechnology Development Co., Pvt. Ltd., Taipei, Taiwan, provided the extracts of* A. camphorata* for this study.

### 2.2. Animals

Male Wistar rats (250–300 g) were used to determine the effects of* A. camphorata* alone or in combination with aspirin against MCAO induced brain damage. Animal care and the general protocols for animal use were approved by the Institutional Animal Care and Use Committee (IACUC) of Taipei Medical University. All animals were clinically normal, free of apparent infection or inflammation, and showed no neurological deficits while they were checked before undergoing the experimental procedures.

### 2.3. MCAO-Induced Ischemia

As demonstrated in our previous studies, an autologous blood clot was administered in rats for MCAO-induced ischemia [13–15]. In brief, 0.6 mL of arterial blood was withdrawn from a femoral catheter by using 1-mL syringe and the blood was immediately injected into PE-10 tubes. The tubes were kept at 4°C for 22 h, and the thread-like clots were removed and placed in a saline-filled dish. The clots were then washed to remove blood cells. Washed clots were transferred to fresh dishes, and the washing process was continued until the saline remained clear. The cleared clot sections were cut into 30 mm long fragments and then drawn up with the saline solution into a PE-10 catheter.

At the time of surgical procedure, animals were anesthetized with a mixture of 75% air and 25% O_2_ gases containing 3% isoflurane. The common carotid artery (CCA) was identified, and approximately 1 cm of the external carotid artery (ECA) was ligated and cut. Consequently, the pterygopalatine artery (PA) was clamped with a 10 mm microaneurysm clamp, and the CCA was similarly clamped before the carotid bifurcation. The internal carotid artery (ICA) was then clamped between the carotid bifurcation and the PA. After that, the PE-50 catheter containing the clot was introduced approximately 5 mm into the previously cut ECA and tied in place with sutures. The ICA clamp was removed, and the clot was flushed into the ICA over a period of approximately 5 s. The PA clamp was removed, and the rat was left in this condition for 1 h.

### 2.4. Experimental Procedure

Rats were randomly separated into six groups at 1 hr after MCA occlusion: (1) a sham-operated group; (2) a group orally treated with an isovolumetric solvent (distilled water) for 60 days, followed by thromboembolic occlusion; (3) and (4) groups orally treated with* A. camphorata* (0.25 and 0.75 g/kg/day) alone for 60 days, followed by thromboembolic occlusion, respectively; (5) and (6) groups treated with* A. camphorata* (0.25 and 0.75 g/kg/day) and aspirin (5 mg/kg/day), followed by thromboembolic occlusion, respectively. An observer blinded to the identity of the groups assessed the neurological deficits after reperfusion by forelimb akinesia test.

### 2.5. Immunoblotting Assay

Expressions of HO-1, iNOS, Bax, and active caspase-3 in the ischemic brain at 24 h after thromboembolic occlusion-reperfusion injury were analyzed by immunoblotting as described by our previous study [14]. Thromboembolic occlusion-insulted and sham-operated rats were anesthetized with chloral hydrate (400 mg/kg, i.p.), and then the apex of the heart was penetrated with a profusion cannula inserted through the left ventricle into the ascending aorta. Perfusion with ice-cold PBS was performed, and an incision was made in the right atrium for venous drainage. Brains were freshly removed and sectioned coronally into four sequential parts from the frontal lobe to the occipital lobe. The third of four parts of the right hemisphere was separately collected, snap-frozen in liquid nitrogen, and stored at −70°C. The frozen tissues were placed in homogenate buffer and homogenized and then sonicated for 10 s three times at 4°C. The sonicated samples were subjected to centrifugation (10,000 ×g).

The supernatant (50 *μ*g protein) was subjected to sodium dodecylsulfate polyacrylamide gel electrophoresis (SDS-PAGE) and electrophoretically transferred to polyvinylidenedifluoride (PVDF) membranes (0.45 *μ*m, Hybond-P, Amersham). After incubation in blocking buffer and being washed three times with TBST buffer (10 mMTris-base, 100 mMNaCl, and 0.1% Tween 20; pH 7.5), blots were treated with an anti-HO-1 polyclonal antibody (pAb, 1 : 1000; R&D, Minneapolis, MN), an anti-iNOS monoclonal antibody (mAb; 1 : 3000, BD Biosciences, San Jose, CA), an anti-BaxpAb (1 : 1000; Cell Signaling, Beverly, MA), and an anti-active caspase-3 pAb (1 : 250; Biovision, Mountain View, CA), or an anti-*α*-tubulin mAb (1 : 2000; Santa Cruz Biotechnology, Santa Cruz, CA) in TBST buffer overnight. Blots were subsequently washed with TBST and incubated with a secondary horseradish peroxidase- (HRP-) conjugated goat anti-mouse mAb or donkey anti-rabbit immunoglobulin G (IgG)(Amersham) for 1 h. Blots were then washed, and the immunoreactive protein was detected using film exposed to enhanced chemiluminescence (ECL) detection reagents (ECL^+^ system; Amersham). The bar graph depicts the ratios of semiquantitative results obtained by scanning reactive bands and quantifying the optical density using video densitometry (Bio-1D vers. 99 image software).

### 2.6. Measurement of Hydroxyl Radical (HO^•^) Formation by Electron Spin Resonance (ESR) Spectrometry

The ESR method used a Bruker EMX ESR spectrometer (Billerica, MA, USA) as described previously [16]. In brief, a Fenton reaction solution (50 *μ*M FeSO_4_ + 2 mM H_2_O_2_) was pretreated with a solvent control (0.1% DMSO) or* A. camphorata* (20 and 40 mg/mL) for 10 min. The rate of hydroxyl radical-scavenging activity was defined by the following equation: inhibition rate = 1 − [signal height (*A. camphorata*)/signal height (solvent control)].

### 2.7. Data Analysis

Experimental results are expressed as the mean ± S.E.M. and are accompanied by the number of observations. The experiments were assessed by the method of analysis of variance (ANOVA). If this analysis indicated significant differences among the group means, then each group was compared using the Newman-Keuls method. A *P* value of <0.05 was considered statistically significant.

## 3. Results

### 3.1. *A. camphorata* Inhibits iNOS and HO-1 Expression in Thromboembolic Cerebral Tissues

**Figure 1:**
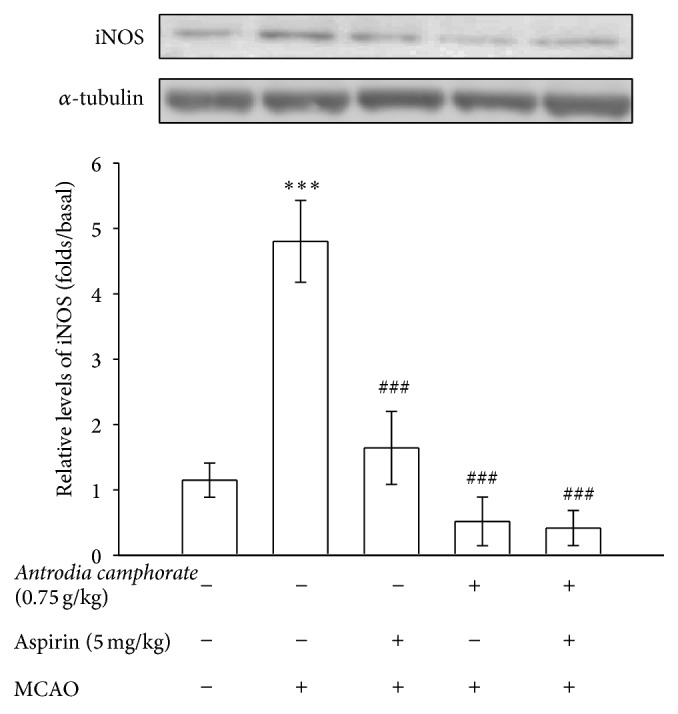
Effects of the extracts of* A. camphorata* combined with aspirin on the expressions of iNOS in cerebral homogenates 24 h after thromboembolic stroke in rats. Fresh brains from each group rats were removed and sectioned coronally into four sequential parts of the frontal lobe to the occipital lobe. The third of four sequential parts of the ischemic-injured hemisphere was separately collected, homogenized, and centrifuged. The supernatant (50 *μ*g protein) was then subjected to SDS-PAGE and transferred onto membranes for analysis of iNOS expressions. Data are presented as the mean ± S.E.M. ^∗∗∗^ *P* < 0.001, compared to the sham-operated group, and ^###^ *P* < 0.001, compared to the MCAO group.

To examine the effect of* A. camphorata* in the ischemic brain, we measured the expression of iNOS and HO-1 in thromboembolic occlusion-insulted cerebral tissues. As shown in Figure 1, iNOS was more evidenced in tissues of thromboembolic occlusion-reperfusion injury than the level obtained in the corresponding area of the sham-operated group. Treatment of* A. camphorata* and aspirin alone at a respective doses of 0.75 g/kg and 5 mg/kg significantly (*P* < 0.001) diminished iNOS expression compared to the MCAO-untreated rats. Moreover, a combined treatment of* A. camphorata* with aspirin apparently potentiated* A. camphorata* mediated suppression of iNOS expression.

**Figure 2:**
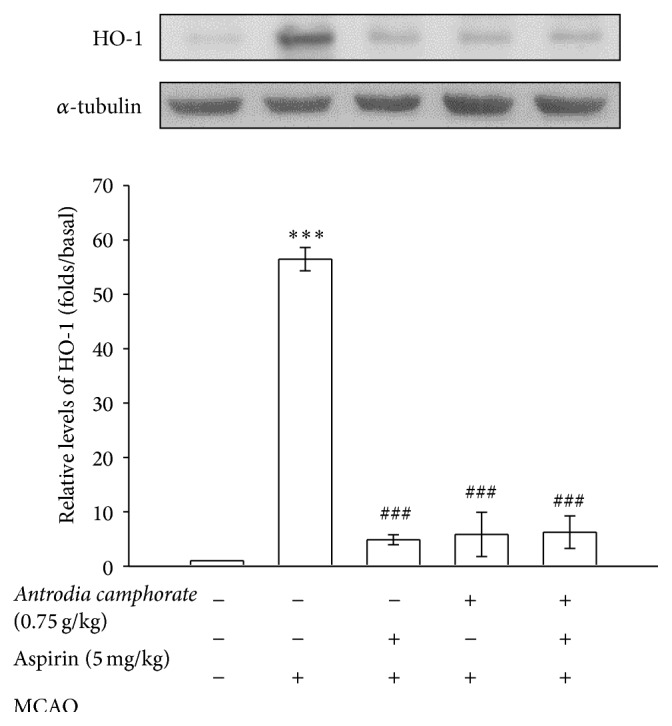
Effects of the extracts of* A. camphorata* combined with aspirin on the expressions of HO-1 in cerebral homogenates 24 h after thromboembolic stroke in rats. Data are presented as the mean ± S.E.M. ^∗∗∗^ *P* < 0.001, compared to the sham-operated group, and ^###^ *P* < 0.001, compared to the MCAO group.

A study has revealed that HO-1 is a key player for drugs upon neuroprotection in transient MCAO model [17]. In this study, Western blot was done to investigate whether* A. camphorata* affects the level of HO-1 expression. The results showed that* A. camphorata* and aspirin alone significantly (*P* < 0.001) reduced the expression of HO-1 protein in brain tissues of MCAO-induced rats (Figure 2). However, this protein expression was not changed when* A. camphorata* was treated with aspirin, since HO-1 expression seemed quite similar as appeared in their individual treatment.

### 3.2. *A. camphorata* Reduces Aspirin-Mediated Suppression of Bax-1 and Active Caspase-3 Expressions in Thromboembolic Cerebral Tissues

**Figure 3:**
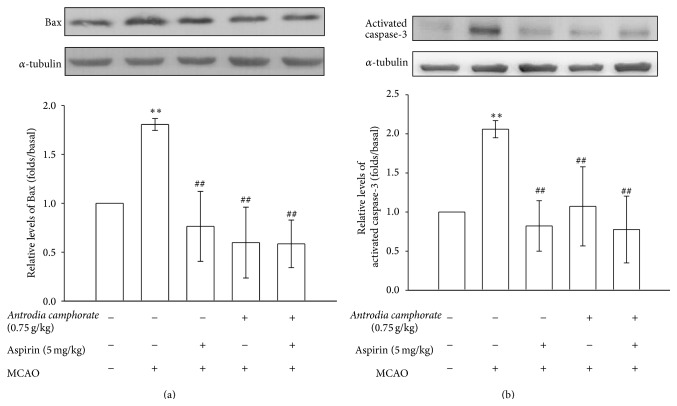
Effects of the extracts of* A. camphorata* combined with aspirin on the expressions of (a) Bax and (b) caspase-3 in cerebral homogenates 24 h after thromboembolic stroke in rats. Data are presented as the mean ± S.E.M. ^∗∗^ *P* < 0.01, compared to the sham-operated group, and ^##^ *P* < 0.01, compared to the MCAO group.

Bax is the proapoptotic member and caspase-3 is the most abundant cysteine protease in the brain and is acutely cleaved and activated in neurons in the early stages of reperfusion, leading to cell apoptosis. In this study, the expression levels of these apoptotic proteins, which are considered as the most important determining factors for the fate of cell and tissues in response to apoptotic stimulations were determined. We found a significant increase in the expressions of Bax (*P* < 0.01) and active caspase-3 (*P* < 0.01) in the injured hemisphere of the MCAO rats as compared to the level obtained in the corresponding area of the sham-operated group (Figures 3(a) and 3(b)). Despite the fact that the individual treatment of aspirin suppresses both the expressions of Bax and activated caspase-3 proteins, the rate of inhibition was potentiated when the treatment was combined with* A. camphorata*.

### 3.3. *A. camphorata* Reduces* In Vitro* OH^•^ Formation

**Figure 4:**
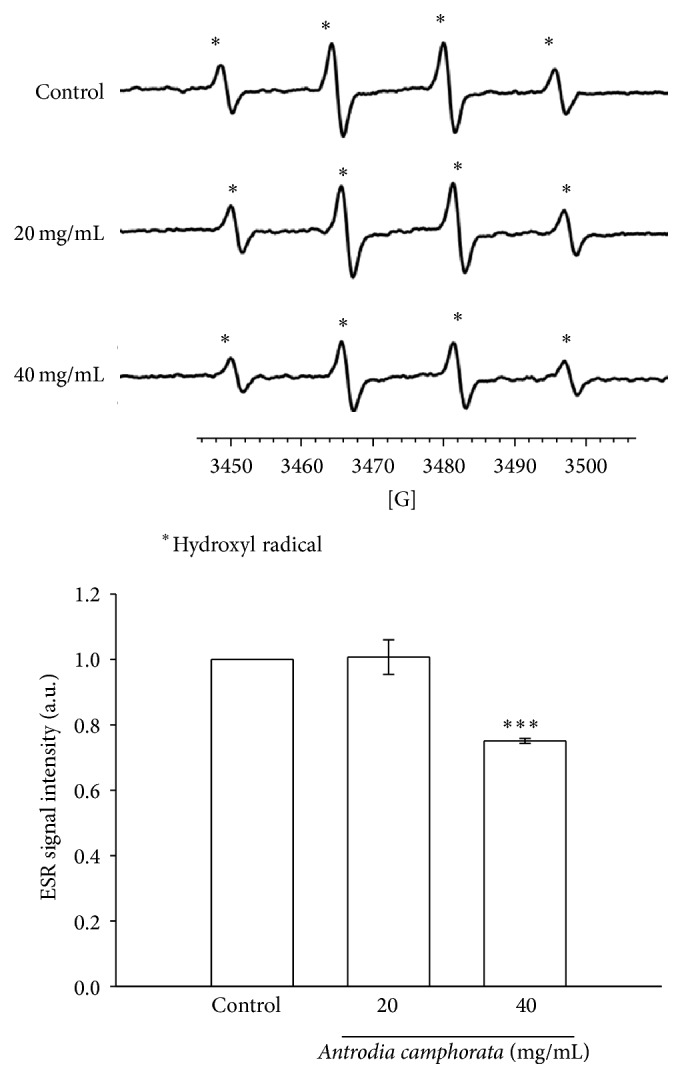
Effects of the extracts of* A. camphorata* on hydroxyl radical formation. ESR spectra show the effects of* A. camphorata* at 40 mg/mL and significantly inhibit hydroxyl radical formation in the fenton reaction. Data are presented as the mean ± S.E.M. ^∗∗∗^ *P* < 0.001, compared to the control group.

To determine the efficacy of* A. camphorata* upon inhibiting fenton reaction-induced OH^•^ formation* in vitro*, a cell-permeative ROS-sensitive dye, DCFDA (nonfluorescent in a reduced state but fluorescent upon oxidation by ROS) was used [16]. In this study, we found that OH^•^ was produced during the fenton reaction very obviously. Interestingly, treatment with* A. camphorata* (40 mg/mL) markedly inhibited the fenton reaction induced OH^•^ (Figure 4); however no effects were observed when* A. camphorata* is treated at a concentration of 20 mg/mL.

## 4. Discussion

Our recent study has demonstrated that* A. camphorata* shows neuroprotective effect against ischemic insults in MCAO model through a mechanism of blood perfusion regulation without increasing hemorrhagic transformation. This treatment also reduced infarct volume in the focal ischemic brain injury and improves neurological outcomes. In this study, we investigated the possible molecular mechanisms of* A. camphorata* on the observed neuroprotective effect. The results were found that an extract of* A. camphorata* possesses neuroprotective effect via antiapoptotic and anti-inflammatory effects and reduces OH radical formation in rat thromboembolic stroke.

Recently, researchers have been attracted to notice the hypothesis that secondary brain damages from hemoglobin as well as its byproducts such as ferrous iron released after heme degradation [18]. Heme or hemin released from hemoglobin accumulates in intracerebral hemorrhage (ICH) [19] and the increased hemin induces HO-1, the rate-limiting enzyme in the oxidative degradation of free heme [20]. High levels of heme metabolites such as ferrous iron resulted in neuronal cell death. Although HO-1 serves a cytoprotective function [21], reports of protective effects of HO-1 inhibitors in experimental ICH models support the idea that HO-1 is a mediator of neurotoxicity in ICH [22, 23] and an attractive therapeutic target for ICH.

In this study, we found that* A. camphorata* exerted neuroprotective effects by reducing the MCAO-induced expression of HO-1. As reported by Chen et al. [24], the induction of HO-1 has been correlated with an experimental model of MCAO and HO-1 knockout mice are reported to be protected from brain injury and functional impairment by ICH [25]. Our results showed that reduced expression of HO-1 by* A. camphorata* protects the MCAO-induced ischemic brain injury. Several reports proposed that a decrease of HO-1 expression by HO-1 inhibitor may provide a protective effect against stroke in various animal models [26, 27]. Recently, Huang et al. reported that treatment of vitamin C offers neuroprotection via reducing HO-1 activity in methamphetamine-induced neurotoxicity in neuronal cells [28]. Combined with the current data, these reports suggest that modulation of HO-1 might have a potential as a new therapy for stroke.

A study demonstrated that iNOS knock-out mice showing reduced brain damage after ischemia, because of an increased expression of iNOS, may also contribute to enhanced neuronal injury [29] and there is an evidence that iNOS plays a role as a mediator in the reduction of infarct size via late preconditioning [30]. A recent study also suggests that iNOS may be involved in the inflammatory reaction that follows cerebral ischemia and iNOS mRNA and enzymatic activity are expressed in brain after permanent MCA occlusion [31]. Treatment with the selective iNOS inhibitor was reported to be reduced infarct volume, suggesting that iNOS activity contributes to ischemic brain damage [32]. A study reported that bioactive constituents of mycelium of* A. camphorata*, antroquinonol B, 4-acetyl-antroquinonol B, 2,3-(methylenedioxy)-6-methylbenzene-1,4-diol, and 2,4-dimethoxy-6-methylbenzene-1,3-diol along with antrodin D inhibit iNOS activity in lipopolysaccharide- (LPS-) activated murine macrophages [33]. In the present study, we demonstrated that treatment of* A. camphorata* in MCAO-induced embolic rats significantly reduced the expression of iNOS, is harmful to the postischemic brain, and may be of worth in the treatment of cerebral ischemia.

Apoptosis is also known as programmed cell death, which is an initiative suicide process after the cells receive a signal or stimulation with some other related gene. The Bcl-2 family proteins are key regulators of apoptosis, which include both antiapoptotic members such as Bcl-2 and the proapoptotic members such as Bax. It has been suggested that a slight change in the dynamic balance of Bcl2/Bax proteins may result either in inhibition or promotion of cell death [34]. Apoptosis has been reported to occur after transient cerebral ischemia and is regulated by the pro- and antiapoptotic proteins and it contributes to ischemic cell damage after stroke [35]. Caspase-3 is an essential protein for brain development, but it also serves as a crucial mediator of neuronal apoptosis [36]. During ischemia, caspase-3 is cleaved and activated whereupon it degrades multiple substrates in the cytoplasm and nucleus leading to cell death [37]. Caspase-3 deficient adult mice reported to be more resistant to ischemic stress both* in vivo* and* in vitro* [37]. Therefore, it is of great interest to control the activation of Bax and caspase-3 for the potential therapeutic treatment of neurological diseases. Several studies have demonstrated that treatment of caspase-3 inhibitors reduced ischemic-induced brain damage [38]. A recent study has suggested that inhibition of Bcl2/Bax ratio may be a novel target for the treatment of stroke [39], and these authors have shown that chemokine-like factor 1 (CKLF1), a novel C-C chemokine, with antibodies displays neuroprotective effects against cerebral ischemia via regulation of apoptosis-related protein expression in ischemic hemisphere. In the present study, it has been shown that* A. camphorata* has neuroprotective effects in MCAO-induced rats via inhibiting Bax and caspase-3 expressions.

Oxidative stress involves the formation of reactive oxygen/nitrogen species (ROS/RNS), which are causal factors in the neuropathology of stroke [40]. Abundant ROS are generated during an acute ischemic stroke through multiple injury mechanisms, such as mitochondrial inhibition, Ca_2_+ overload and reperfusion injury [41]. Brain ischemia generates super oxide radical (O_2_^•^), from which H_2_O_2_ is formed. H_2_O_2_ is the source of hydroxyl radical (OH^•^). An* in vivo* study has revealed that a dry matter of fermented filtrate (DMF) from* A. camphorata* in submerged culture shows antioxidant like effects against H_2_O_2_-induced cytotoxicity in HepG2 and carbon tetrachloride- (CCl_4_-) induced hepatotoxicity [11]. They showed that DMF may play a role in preventing oxidative damage in living systems by upregulating hepatic glutathione-dependent enzymes to preserve the normal reduced and oxidized glutathione (GSH/GSSH) ratio and scavenging free radicals formed during CCl_4_ metabolism.

A previous study was reported that polysaccharides extracted from fruiting bodies or cultured mycelia of* A. camphorata* exhibit an antihepatitis B virus effect [42]. In that study, the authors have specified that extracts from cultured mycelia of* A. camphorata* inhibit N-formyl-methionyl-leucyl-phenylalanine (fMLP) or phorbol 12-myristate 13-acetate- (PMA-) induced ROS production in peripheral human neutrophils (PMN) or mononuclear cells (MNC). OH^•^ can be produced from O_2_ under a variety of stress conditions and are involved in numerous cellular disorders such as inflammations, embryo teratogenesis, herbicide effects, cell death, and killing of microorganisms in pathogen-defense reactions. It is generally assumed that OH^•^ is generated in biological systems from H_2_O_2_ by the Fenton reaction [43, 44]. Therefore, in the present study, we used fenton reaction to evaluate the hydroxyl radical-scavenging activity of* A. camphorata* by the ESR experiment. We found that* A. camphorata* significantly inhibits OH^•^ formation at a higher concentration of 40 mg/mL. These results proposed that neuroprotection by* A. camphorata* may be involved, at least partly, in the inhibition of free radical formation.

In conclusion, our recent study was demonstrated that* A. camphorata* provides neuroprotection against MCAO-induced ischemic stroke via improved neurological functional scores and reduced infarct volume without causing hemorrhagic incidence when it is used in conjunction with aspirin therapy; nevertheless, the mechanisms underlying remained intricate. Therefore, we performed this study and found that the neuroprotective effect of* A. camphorata* is possibly via enhanced inhibition of HO-1, followed by the inhibition of inflammatory responses (i.e., iNOS) and apoptosis (Bax and activated caspase-3) in the ischemic brain. In addition, neuroprotection by* A. camphorata* may be involved, at least partly, by the inhibition of free radical formation.
